# PRISMA—Efficacy and Safety of Vedolizumab for Inflammatory Bowel Diseases: A Systematic Review and Meta-Analysis of Randomized Controlled Trials

**DOI:** 10.1097/MD.0000000000000326

**Published:** 2014-12-02

**Authors:** Man Cai Wang, Ling Yi Zhang, Wei Han, Yuan Shao, Mo Chen, Rui Ni, Gen Nian Wang, Feng Xian Wei, Ya Wu Zhang, Xiao Dong Xu, You Cheng Zhang

**Affiliations:** From the Department of General Surgery (MCW, WH, YS, MC, RN, GNW, FXW, YWZ, XDX, YCZ); Hepato-Biliary-Pancreatic Institute, Lanzhou University Second Hospital (MCW, WH, YS, MC, RN, GNW, FXW, YWZ, XDX, YCZ); and Gansu Provincial-Level Key Laboratory of Digestive System Tumors (MCW, LYZ, WH, YS, MC, RN, GNW, FXW, YWZ, XDX, YCZ); and Department of Hepatology, Lanzhou University Second Hospital, Lanzhou, China (LYZ).

## Abstract

Vedolizumab is an anti-inflammatory monoclonal antibody that exclusively targets the α4β7 integrin. We aimed to systematically review the efficacy and safety of vedolizumab for patients with inflammatory bowel diseases (IBDs).

PubMed, EMBASE, and the Cochrane Library were searched up to May 2014. Randomized controlled trials examining the efficacy or safety of vedolizumab in patients with IBDs were eligible for inclusion. Data were extracted independently by 2 investigators and pooled using Review Manager 5.0 software (The Cochrane Collaboration, Copenhagen). Results were expressed as the relative risk (RR) with 95% confidence intervals (CIs).

Six randomized controlled trials involving 2815 patients were eligible for inclusion. Vedolizumab was more effective than placebo for patients with ulcerative colitis and Crohn disease (CD) in clinical response (RR = 1.82, 95% CI, [1.43, 2.31]; RR = 1.46, 95% CI [1.18,1.81]) and clinical remission (RR = 2.23, 95% CI [1.35, 3.68]; RR = 1.71, 95% CI [1.25, 2.34]) during induction therapy. A superior effect was found during maintenance therapy in durable clinical/CD Activity Index-100 response (RR = 2.22, 95% CI [1.62, 3.05]; RR = 1.48, 95% CI [1.13, 1.94]) and clinical remission (RR = 2.55, 95% CI [1.38, 4.70]; RR = 1.15, 95% CI [0.75, 1.77]). However, vedolizumab may be associated with serious adverse events (RR = 1.25, 95% CI [1.03, 1.52]) and nasopharyngitis (RR = 1.56, 95% CI [1.08, 2.25]) for patients with CD.

Vedolizumab was more effective than placebo as induction and maintenance therapy for IBDs, with an acceptable short-term safety profile, and achieving cure, although it may be associated with serious adverse events and nasopharyngitis for patients with CD.

## Abstract 2

Supplemental Digital Content is available in the text

## INTRODUCTION

Inflammatory bowel diseases (IBDs), primarily including ulcerative colitis (UC) and Crohn disease (CD), are chronic inflammatory disorders of the gastrointestinal (GI) tract.^1,2^ The incidence and prevalence of IBD are increasing over time globally.^3,4^

Current medical treatment modalities for IBD include 5-aminosalicylates, corticosteroids, immunosuppressants, and biologic therapy.^5–7^ Surgery is often indicated for severe disease or serious complications.^1^ Although these drugs are effective and have acceptable side effects, many patients do not have a clinical response and corticosteroids become necessary.^8^ In fact, corticosteroid therapy is effective but is frequently associated with serious adverse effects.^6,9^ In addition, drug dependency and resistance are produced in approximately 20% to 40% of IBD patients despite the use of immunosuppressant drugs in an attempt to reduce corticosteroid requirements.^10^ A meta-analysis of immunosuppressive therapy for IBD showed no statistically significant benefit in inducing remission in active CD and UC compared with placebo.^11^ Antitumor necrosis factor (TNF) agents such as infliximab, adalimumab, and certolizumab pegol have dramatically improved IBD treatment. However, a significant proportion of patients with UC and CD will not respond or lose response to these agents over time. Anti-TNF agents are also associated with complications.^12–16^

There are many theories on the pathogenesis of IBD, all of which ultimately attribute leukocytic infiltration of the intestinal mucosa and a disorder of intestinal barrier function.^1^ Thus, inhibition of leukocyte trafficking to the gut mucosa has become an important target for the development of IBD drugs.^17–19^ Natalizumab, the first antagonist of leukocyte trafficking, targets the α4β7 and α4β1 integrins that control leukocyte adhesion to the vascular endothelium.^17^ Although it has been shown to be effective in induction therapy for patients with moderately to severely active CD,^20–23^ its large-scale use was limited because of the potential for progressive multifocal leukoencephalopathy (PML), a fatal demyelinating disease of the central nervous system.^24,25^

Vedolizumab was designed specifically to inhibit gut α4β7 integrins; preliminary results have shown vedolizumab to be potentially effective for patients with active CD and UC.^26–34^ The uncertainty of adverse events was presented during those studies. Our study is the first to systematically review the efficacy of vedolizumab for patients with IBD.

## METHODS

### Search Strategy and Study Selection

An electronic search was conducted using MEDLINE, EMBASE, and the Cochrane library up to May 2014. The search strategy was not limited by language. Search terms (both free text and medical subject headings) included: “inflammatory bowel diseases,” “ulcerative colitis,” “Crohn's disease,” “vedolizumab,” “MLN0002,” “MLN02,” and “LDP-02.” Studies were assessed independently by 2 investigators; eligibility criteria are shown in Table 1.

**TABLE 1:**
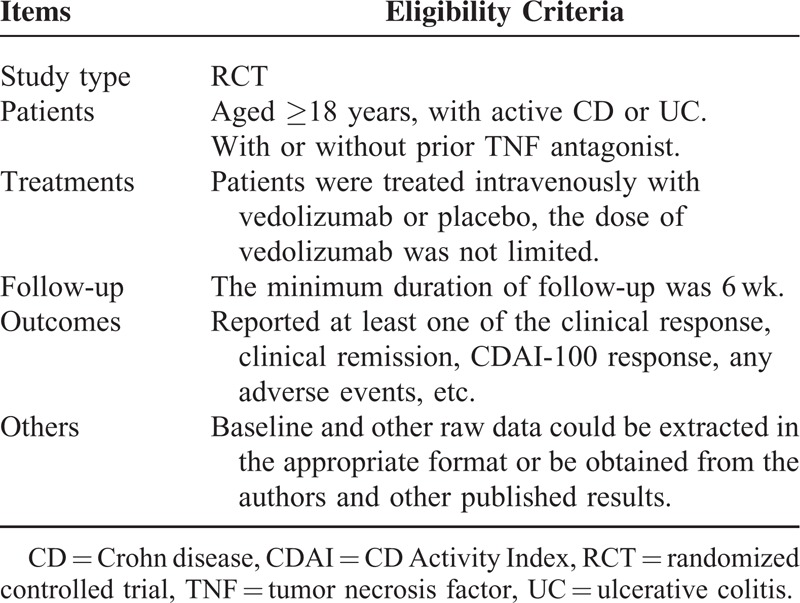
Eligibility Criteria of the Systematic Review and Meta-Analysis

### Data Extraction

Data were carefully extracted by 2 independent investigators according to the inclusion criteria in a prespecified Microsoft Excel spreadsheet. Disagreement was resolved by discussion with a third reviewer. The following data were collected for each study: demographic data of trial participants (age, sex), number of patients, country of origin, number of centers, dosage and schedule of vedolizumab, and duration of follow-up. Primary outcomes included clinical remission and clinical or CD Activity Index (CDAI)-100 response in induction and maintenance therapy. Clinical response was used to evaluate the effect of vedolizumab for patients with UC, and CDAI-100 response was used for patients with CD.

In addition, data about adverse events were extracted for each study. Data were extracted as intention-to-treat analyses in which all dropouts or missing data were considered to be treatment failures

### Risk of Bias Assessment

Two investigators independently evaluated the methodologic quality of the studies according to the Cochrane Risk of Bias Tool for RCTs;^35^ differences were resolved by discussion with a third investigator. Six components were used, including adequate sequence generation, allocation concealment, blinding, incomplete outcome data addressed, free of selective reporting, and free of other bias.

### Statistical Analysis

Data were pooled using Review Manager 5.0 software (RevMan 5.0). All data were analyzed on an intention-to-treat basis. Efficacy and safety were analyzed using dichotomous data, and the results were expressed as relative risk (RR) with 95% confidence intervals (CIs). A random effects model was used to give a more conservative estimate of effect and adverse events. Clinical heterogeneity was assessed by examining the characteristics of the included studies, whereas statistical heterogeneity was assessed using the *I*^*2*^ statistic with a cutoff of 50%, and the χ^2^ test^36^ with a *P* value <0.10. Subgroup analyses were performed to identify the different effects of vedolizumab for UC and CD. Finally, all outcomes were reanalyzed using a fixed effects model to estimate the stability of the meta-analysis.

## RESULTS

### Literature Search Results

**FIGURE 1:**
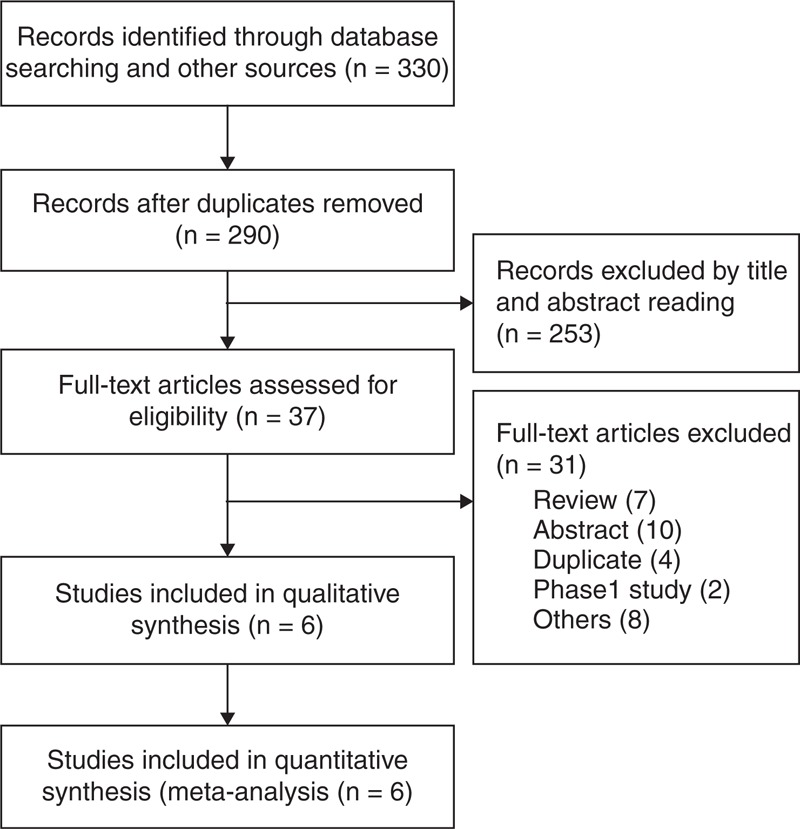
Search flow diagram.

**FIGURE 2:**
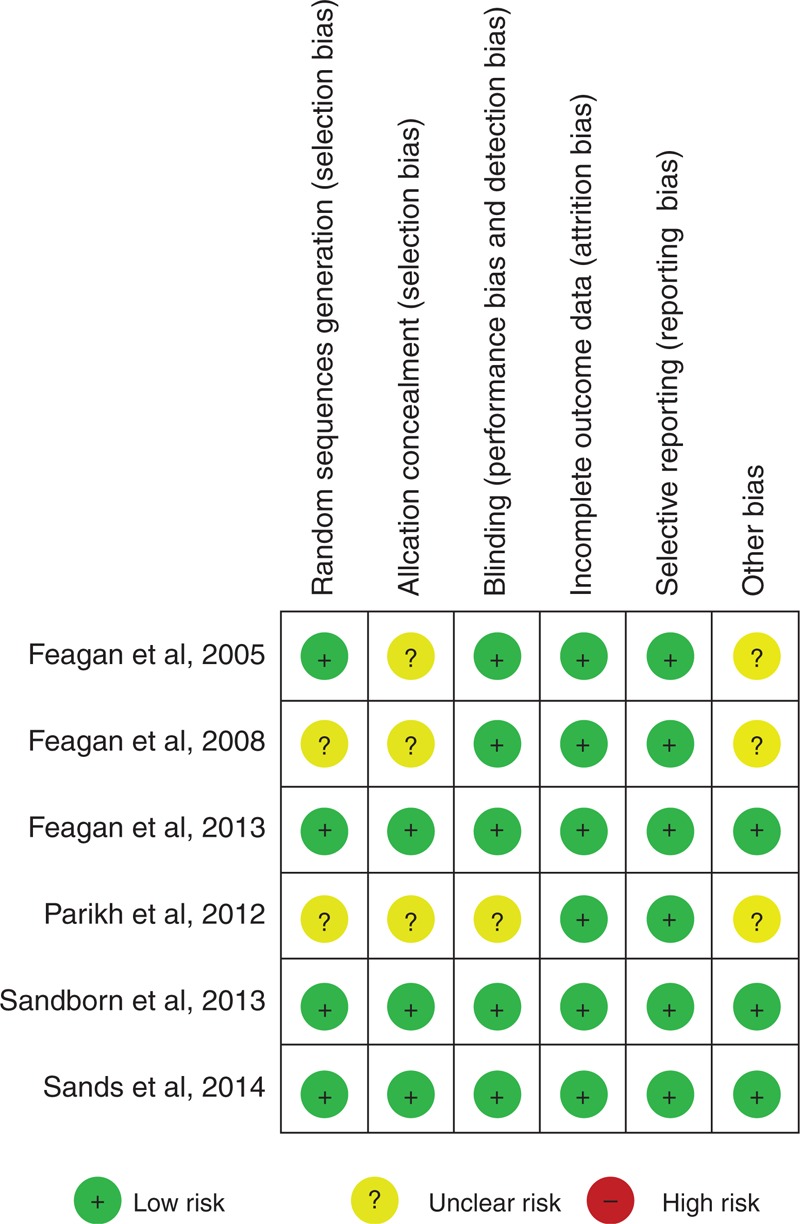
Outcome of risk of bias.

We identified 6 eligible RCTs,^29–34^ evaluating the effect and adverse events of vedolizumab in 2815 patients with active IBD. The search flow diagram is shown in Figure 1. One RCT^28^ was excluded because of no placebo group. Table 2 presents the clinical characteristics of the included studies. There was no clinical heterogeneity found in these studies. All studies were conducted at multiple medical centers, vedolizumab or placebo was given intravenously (at dosages of 0.5, 2, 6, 10 mg/kg, or 300 mg), and the duration of follow-up ranged from 42 days to 52 weeks. Randomization was computer-generated in 4 trials^29,32–34^ and allocation concealment was performed at a central location in 2 trials;^30,31^ only 1 trial^31^ did not report blinding. Therefore, the risk of bias was low (Figure 2).

**TABLE 2:**
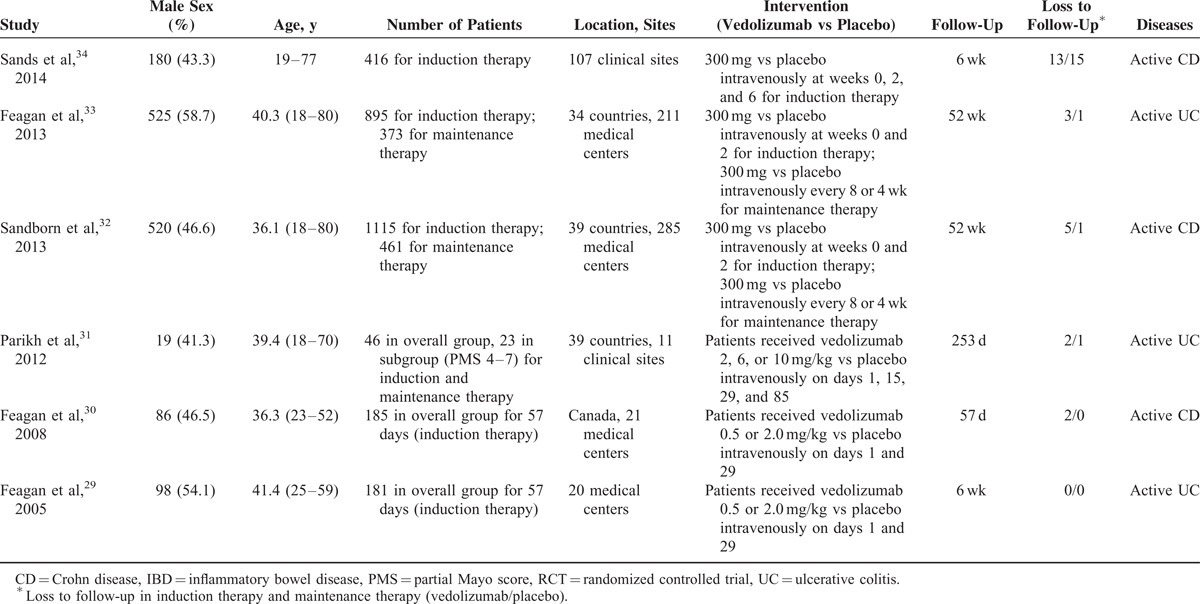
Characteristics of RCTs of Vedolizumab Versus Placebo for Active IBD

## EFFICACY OF VEDOLIZUMAB FOR IBD

### Clinical Remission in Induction Therapy

**FIGURE 3:**
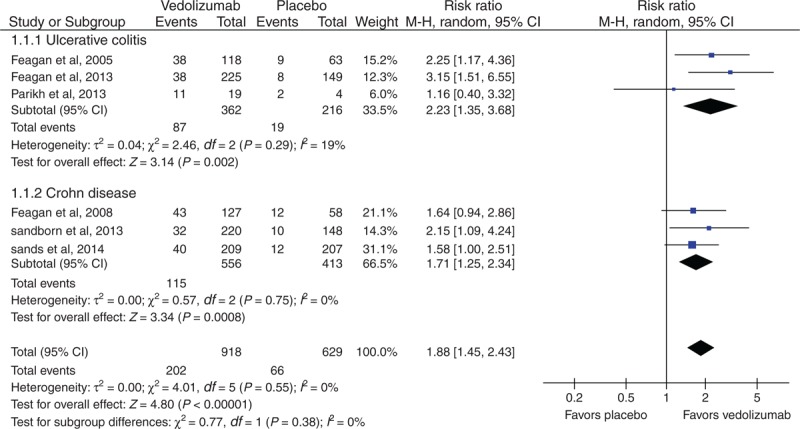
Meta-analysis result of clinical remission in induction therapy. CI = confidence interval.

Six RCTs^29–34^ evaluated clinical remission in induction therapy, comparing vedolizumab with placebo, which was reported on days 42,^29,32–34^ 43,^31^ and 57,^30^ respectively. No heterogeneity existed between these studies (*I*^*2*^ = 0%, *P* = 0.55). In the overall analysis, there was a statistically significant difference between the vedolizumab and placebo groups (RR = 1.88; 95% CI [1.45, 2.43]). Subgroup analysis was performed to assess the different effects of vedolizumab for UC and CD patients. The respective results were similar to the overall analysis (Figure 3).

### Clinical or CDAI-100 Response in Induction Therapy

**FIGURE 4:**
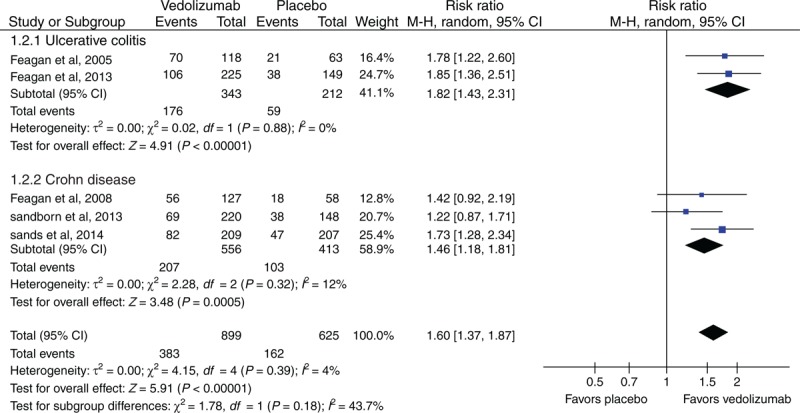
Meta-analysis result of clinical or CDAI-100 response in induction therapy. CI = confidence interval, CDAI = Crohn Disease Activity Index.

Two RCTs^29,33^ reported the clinical response for UC patients, and 3 RCTs^30,32,34^ reported the CDAI-100 response for CD patients. Heterogeneity existed between these studies (*I*^*2*^ = 43.7%, *P* = 0.18). Our meta-analysis showed that there was a statistically significant difference between vedolizumab and placebo groups for UC patients (RR = 1.82; 95% CI [1.43, 2.31]) as well as CD patients (RR = 1.46; 95% CI [1.18, 1.81]) (Figure 4).

### Clinical Remission in Maintenance Therapy

Three RCTs^31–33^ assessed clinical remission for patients with active IBD in maintenance therapy. Clinical remission was reported on days^32,33^ 364 and^31^ 253. There was a statistically significant difference on comparing vedolizumab with placebo groups (RR = 2.06; 95% CI [1.47, 2.88]). Results in the UC and CD subgroups were similar to the overall analysis (Table 3).

**TABLE 3:**
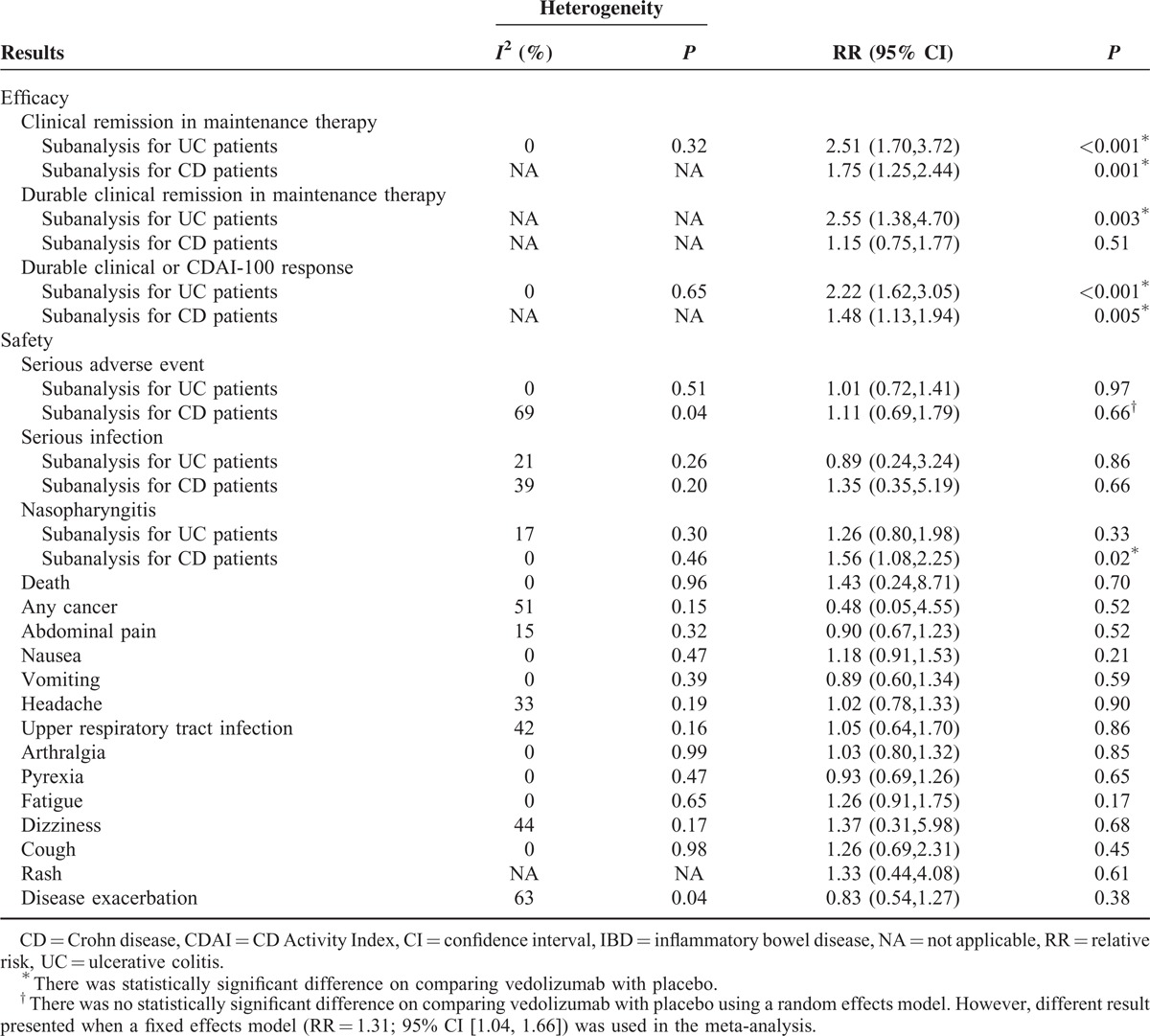
Efficacy and Safety of Vedolizumab Versus Placebo for IBD in the Systematic Review

Two studies^32,33^ reported durable clinical remission. There was significant heterogeneity among these studies (*I*^*2*^ = 78, *P* = 0.03). In the overall analysis, there was no statistically significant difference on comparing vedolizumab with placebo groups (RR = 1.66; 95% CI [0.76, 3.64]). However, a statistically significant difference existed for UC patients in the subanalysis (Table 3).

### Durable Clinical or CDAI-100 Response

Two studies^31,33^ assessed durable clinical response for patients with UC, and one study^32^ assessed the durable CDAI-100 response for patients with CD. Heterogeneity existed among these studies (*I*^*2*^ = 49%, *P* = 0.14). The overall analysis demonstrated that there was a statistically significant difference on comparing vedolizumab with placebo groups (RR = 1.81; 95% CI [1.29, 2.52]). Results in UC and CD subgroups were similar to the overall analysis (Table 3).

## SAFETY OF VEDOLIZUMAB FOR IBD

### Serious Adverse Events

All of the included RCTs^29–34^ reported serious adverse events during the follow-up period. There was heterogeneity among these studies (*I*^*2*^ = 45%, *P* = 0.22). The meta-analysis showed that no statistically significant difference existed between vedolizumab and placebo groups (RR = 1.21; 95% CI [1.00, 1.46]) (Table 3).

As one of the serious adverse events, serious infection was reported in 5 RCTs.^29,30,32–34^ There was no statistically significant difference on comparing vedolizumab with placebo groups (RR = 1.17; 95% CI [0.51, 2.69]) (Table 3).

### Nasopharyngitis

All of the included RCTs^29–34^ reported the adverse event of nasopharyngitis during the follow-up period. According to the overall analysis, a statistically significant difference existed between vedolizumab and placebo groups (RR = 1.42; 95% CI [1.09, 1.83]). However, there was no significant difference for UC patients in the subanalysis (Table 3).

### Other Adverse Events

Other adverse events included PML, death, cancer, abdominal pain, nausea, vomiting, headache, upper respiratory tract infection, arthralgia, pyrexia, and fatigue. There was no statistically significant difference for any of these adverse events on comparing vedolizumab with placebo groups (Table 3). All of the adverse events in each included study are shown in Table S1, http://links.lww.com/MD/A123.

### Sensitivity Analysis

All of the outcomes were reanalyzed using a fixed effects model to estimate the stability of this meta-analysis. Most of the results were consistent with these described above, except for serious adverse events in the CD subanalysis (RR = 1.31; 95% CI [1.04, 1.66]).

## DISCUSSION

IBDs are chronic GI tract diseases characterized by an exacerbated inflammatory cell infiltrate in the gut mucosal tissue.^37^ Multiple inflammatory cell types participate in the pathogenesis of IBD, of which lymphocytes play a central role in the chronic inflammatory process.^37^ Lymphocytes migration and adhesion to specific tissues are determined by the combination of receptors rather than a single receptor or adhesive molecule.^38^ Data indicate that CCR9 is required for T-cell migration and localization in the small bowel, whereas α4β7 is required for T-cell migration and localization in the small bowel and colon.^39^

Vedolizumab (previous versions were known as MLN02, LDP02, and MLN0002), a gut-selective anti-inflammatory biologic agent, targets the α4β7 integrin exclusively and antagonizes its interaction with mucosal addressin cell adhesion molecule-1 (MAdCAM-1).^26^ The pharmacokinetics and pharmacodynamics of vedolizumab were studied in healthy individuals and patients with UC or CD over a dose range of 2 to 10 mg/kg. Pharmacodynamic studies suggested that as soon as the serum vedolizumab concentration decreased below the limit of detection of the assay, α4β7 integrin\MAdCAM-1-mediated trafficking was restored.^40^ Previous studies demonstrated that no further changes in these parameters occurred at dose levels >2 mg/kg; thus, the saturation of a rapid elimination process was at low concentrations.^40^

Vedolizumab still shows benefit during induction and maintenance therapy in this meta-analysis, although it failed to meet the primary endpoints of GEMINI II and GEMINI III. Vedolizumab could increase clinical response and clinical remission for patients with active UC, as well as the CDAI-100 response and clinical remission for patients with active CD in both induction and maintenance therapy. Furthermore, Sands et al^34^ demonstrated that greater proportions of vedolizumab-treated patients compared with placebo-treated patients were in clinical remission at week 10 in the TNF antagonist failure population. Outcomes in this meta-analysis are consistent with those in the included studies with regard to UC; interestingly, 2 studies^30,32^ about CD showed that vedolizumab was not more effective than placebo in inducing the CDAI-100 response.

PML is one of the main safety focuses for vedolizumab use. As one of the selective adhesion molecule inhibitors, natalizumab binds to the α4 subunit of both α4β1 and α4β7 integrins, and antagonizes their interaction with all known ligands.^17^ It was approved in 2008 for the treatment of CD,^23^ but its large-scale use was limited because of PML.^24,25^ Over the past 7 years, >3000 patients have been exposed to vedolizumab, and no cases of PML have been observed.^40^ This can be explained by the relative gut selectivity of vedolizumab in antagonizing α4β7–MAdCAM-1 interactions.^29–34^

There is a concern that, compared with placebo in this meta-analysis, vedolizumab is associated with a higher rate of serious adverse events (21.7% vs 14.3%) for patients with CD. This is similar to the result of Sandborn et al^32^ (24.4% vs 15.3%). Furthermore, nasopharyngitis occurs more frequently with vedolizumab than with placebo (11.1% vs 6.2%) for CD patients during the follow-up period. However, the fact that vedolizumab is not associated with any adverse events for UC patients is confusing. One hypothesis is that CD may represent a more systemic disorder as it could affect any portion of the GI system, from mouth to anus, and is characterized by transmural inflammation, fistulas, and multiorgan involvement.^17^

It is noteworthy that definitions of clinical response and clinical remission are not all consistent among these included studies (Table S2, http://links.lww.com/MD/A123), which may affect the judgment of vedolizumab for patients with UC. Also, most of the included RCTs report neither the specific details regarding serious adverse events nor the medical care impact of those complications. We can conclude from the small loss-to-follow-up rate in the 6 RCTs that most of the adverse events are acceptable. Rare adverse events can only be identified after exposing a large number of patients during Phase IV studies. Another deficiency is the limited number of RCTs, which may partly impact the overall conclusion.

In conclusion, vedolizumab was more effective than placebo as induction and maintenance therapy for IBD, with an acceptable short-term safety profile and cure rate. However, it is necessary to perform a reanalysis when more data become available.
